# Using ChatGPT to evaluate cancer myths and misconceptions: artificial intelligence and cancer information

**DOI:** 10.1093/jncics/pkad015

**Published:** 2023-03-17

**Authors:** Skyler B Johnson, Andy J King, Echo L Warner, Sanjay Aneja, Benjamin H Kann, Carma L Bylund

**Affiliations:** Department of Radiation Oncology, University of Utah School of Medicine, Huntsman Cancer Institute, Salt Lake City, UT, USA; Cancer Control and Population Sciences, Huntsman Cancer Institute, Salt Lake City, UT, USA; Cancer Control and Population Sciences, Huntsman Cancer Institute, Salt Lake City, UT, USA; Department of Communication, University of Utah, Salt Lake City, UT, USA; Cancer Control and Population Sciences, Huntsman Cancer Institute, Salt Lake City, UT, USA; College of Nursing, University of Utah, Salt Lake City, UT, USA; Center for Outcomes Research and Evaluation, Yale School of Medicine, New Haven, CT, USA; Department of Therapeutic Radiology, Yale School of Medicine, New Haven, CT, USA; Department of Radiation Oncology, Dana-Farber Cancer Institute/Brigham and Women’s Hospital, Harvard Medical School, Boston, MA, USA; Department of Health Outcomes and Biomedical Informatics, University of Florida College of Medicine, Gainesville, FL, USA

## Abstract

Data about the quality of cancer information that chatbots and other artificial intelligence systems provide are limited. Here, we evaluate the accuracy of cancer information on ChatGPT compared with the National Cancer Institute’s (NCI’s) answers by using the questions on the “Common Cancer Myths and Misconceptions” web page. The NCI’s answers and ChatGPT answers to each question were blinded, and then evaluated for accuracy (accurate: yes vs no). Ratings were evaluated independently for each question, and then compared between the blinded NCI and ChatGPT answers. Additionally, word count and Flesch-Kincaid readability grade level for each individual response were evaluated. Following expert review, the percentage of overall agreement for accuracy was 100% for NCI answers and 96.9% for ChatGPT outputs for questions 1 through 13 (ĸ = ‒0.03, standard error = 0.08). There were few noticeable differences in the number of words or the readability of the answers from NCI or ChatGPT. Overall, the results suggest that ChatGPT provides accurate information about common cancer myths and misconceptions.

Patients are increasingly turning to the internet for information about cancer, with 80% of US adults reportedly using the internet to seek health information (1). Within the online communication environment, cancer misinformation and harmful information remain a serious concern (2). Chatbots and other artificial intelligence (AI) systems have become increasingly popular in recent years for providing information and assistance to users in online spaces (3); recently, 1 such AI system receiving a lot of public and news attention is ChatGPT, which uses natural language processing to generate responses to user input. Given the importance of accurate information in the field of cancer research and treatment, determining the accuracy of AI (mis)information outputs from chat platforms such as ChatGPT is critical to clinicians and, more broadly, health and medical communicators. As an initial step in monitoring these public platforms, we engaged in a small-scale study of the information ChatGPT generated.

Using the National Cancer Institute’s (NCI’s) web page “Common Cancer Myths and Misconceptions” (4), on December 20, 2022, we asked ChatGPT (December 15, 2022, version) 13 questions about cancer that are common points of confusion among the public (per NCI). The NCI’s answers and ChatGPT answers to each question were blinded, and then evaluated for accuracy based on the established knowledge of 5 scientific reviewers with expertise in cancer treatment and cancer misinformation (B.K., C.B., A.K., E.W., and S.A.) between December 20, 2022, and January 3, 2023. Expert reviewers were not compensated. Accuracy ratings (yes vs no) were evaluated independently for each question, and then compared between the blinded NCI and ChatGPT answers. Interrater agreement was evaluated by Fleiss ĸ (5). Statistical analyses were performed using Stata, version 17 (StataCorp LP).

Following expert review, 13 of 13 NCI answers were rated as accurate by 5 of 5 expert reviewers, demonstrating 100% interrater agreement. ChatGPT outputs were determined to be accurate by 5 of 5 reviewers for 11 of 13 questions, demonstrating 96.9% overall agreement. The remaining 2 questions (question 5, “Does my attitude—positive or negative—determine my risk of getting or surviving cancer?” and question 9, “Do power lines cause cancer?”) were rated as accurate by 4 of 5 reviewers, with a single unique reviewer rating each question as inaccurate (ĸ = ‒0.03, SE = 0.08, *P* = .6). Table 1 displays all questions and associated answers from NCI and ChatGPT, respectively, as well as word count and Flesch-Kincaid readability grade level for each individual response. There were few noticeable differences in the number of words or the readability of the answers from NCI or ChatGPT. Answers from both sources were written at a less readable rate than would likely be recommended by health literacy advocates, for example. Although our team did not code for specific message features, multiple observers noted their perception that the ChatGPT messages often used terms associated with hedging and uncertainty that could lead to the adoption of a belief or practice that could be harmful. Overall, the results suggest that output from ChatGPT provides accurate information about common cancer myths and misconceptions without providing misinformation or harmful information to users. To evaluate whether repeated questioning could result in outputs with semantic variations that could lead to misinformation, we completed 5 runs repeatedly from the same input for each of the 13 questions (Supplementary Table 1, available online). On review, although subtle sentence structure changes were noted, the answers remained consistent, suggesting that answers remain accurate with repetitive questioning.

**Table 1.** Cancer myths and misconceptions questions, answers, and descriptive information^a^

Question	NCI answer	NCI WC	NCI FKG	ChatGPT answer	C WC	C FKG
*Is cancer a death sentence?*	In the United States, the likelihood of dying from cancer has dropped steadily since the 1990s. Five-year survival rates for some cancers, such as breast, prostate, and thyroid cancers, now are 90% or better. The 5-year survival rate for all cancers combined is currently about 68%. For more information, see the Annual Report to the Nation on the Status of Cancer. It is important to note, however, that these rates are based on data from large numbers of people. How long an individual cancer patient will live and whether he or she will die from the disease depend on many factors, including whether the cancer is slow or fast growing, how much the cancer has spread in the body, whether effective treatments are available, the person’s overall health, and more.	133	15.8	Cancer is not necessarily a death sentence. The prognosis for people with cancer depends on many factors, including the type and stage of the cancer, the person’s overall health, and the effectiveness of treatment. Some types of cancer can be cured, while others may be managed through treatments such as surgery, chemotherapy, radiation, or targeted therapies. In some cases, people with cancer may live many years with the disease, while in other cases, cancer may be more aggressive and may progress quickly. It is important to work with a healthcare team to determine the best treatment plan based on the specifics of your situation.	104	11.4
*Will eating sugar make my cancer worse?*	No. Although research has shown that cancer cells consume more sugar (glucose) than normal cells, no studies have shown that eating sugar will make your cancer worse or that, if you stop eating sugar, your cancer will shrink or disappear. However, a high-sugar diet may contribute to excess weight gain, and obesity is associated with an increased risk of developing several types of cancer. For more information, see the NCI fact sheet on Obesity and Cancer.	76	12.7	There is no scientific evidence to support the idea that eating sugar can directly cause cancer or make it worse. However, there is some evidence to suggest that high sugar intake may be associated with an increased risk of certain types of cancer, such as breast and colon cancer. This is because a diet high in sugar is often associated with being overweight or obese, and carrying excess weight has been linked to an increased risk of cancer. Additionally, high sugar intake may be linked to inflammation in the body, which has also been associated with an increased risk of cancer. However, it is important to note that the link between sugar intake and cancer risk is still being studied, and more research is needed to understand the relationship between these factors. It is generally recommended to limit your intake of added sugars, such as those found in sugary drinks, sweets, and baked goods, as part of a healthy diet. This is because a diet high in added sugars has been linked to an increased risk of several health problems, including tooth decay, type 2 diabetes, and heart disease. Instead of sugary foods, it is important to focus on eating a diet that is rich in a variety of vegetables, fruits, whole grains, and lean protein sources. This can help you maintain a healthy weight and reduce your risk of chronic diseases, including cancer.	234	13.7
*Do artificial sweeteners cause cancer?*	No. Researchers have conducted studies on the safety of the artificial sweeteners (sugar substitutes) saccharin (Sweet 'N Low^®^, Sweet Twin^®^, NectaSweet^®^); cyclamate; aspartame (Equal^®^, NutraSweet^®^); acesulfame potassium (Sunett^®^, Sweet One^®^); sucralose (Splenda^®^); and neotame and found no evidence that they cause cancer in humans. All of these artificial sweeteners except for cyclamate have been approved by the Food and Drug Administration for sale in the United States. For more information, see the NCI fact sheet on Artificial Sweeteners and Cancer.	80	9.5	There is no clear evidence that artificial sweeteners cause cancer. Some studies have suggested a link between the use of artificial sweeteners and an increased risk of certain types of cancer, while other studies have not found such a link. The results of these studies have been mixed, and more research is needed to understand the relationship between artificial sweeteners and cancer. The U.S. Food and Drug Administration (FDA) has approved several artificial sweeteners as safe for consumption, including aspartame, saccharin, and sucralose. These sweeteners have been extensively studied and have been found to be safe for human consumption at the levels typically consumed. However, it is important to note that some people may be sensitive to certain artificial sweeteners and may experience side effects such as headaches, digestive issues, or allergic reactions. If you are concerned about the safety of artificial sweeteners, it is always a good idea to talk to your healthcare provider for guidance.	157	14.9
*Is cancer contagious?*	In general, no. Cancer is not a contagious disease that easily spreads from person to person. The only situation in which cancer can spread from one person to another is in the case of organ or tissue transplantation. A person who receives an organ or tissue from a donor who had cancer in the past may be at increased risk of developing a transplant-related cancer in the future. However, that risk is extremely low—about two cases of cancer per 10,000 organ transplants. Doctors avoid the use of organs or tissue from donors who have a history of cancer. In some people, cancers may be caused by certain viruses (some types of human papillomavirus, or HPV, for example) and bacteria (such as *Helicobacter pylori*). While a virus or bacterium can spread from person to person, the cancers they sometimes cause cannot spread from person to person. For more information about cancer-causing viruses and bacteria, see the NCI fact sheet on *Helicobacter pylori* and Cancer and our pages on HPV and Cancer and Infectious Agents.	174	13.9	Cancer is not a contagious disease, meaning it cannot be transmitted from person to person like a cold or the flu. Cancer is a disease in which abnormal cells divide and grow uncontrollably, forming tumors. There are many different types of cancer, and each type can have a different cause. Some types of cancer are caused by genetic factors, while others may be caused by exposure to certain substances, such as tobacco smoke or radiation. Some types of cancer may also be caused by infections, such as the human papillomavirus (HPV), which can cause certain types of cancer, including cervical cancer and some types of head and neck cancer. However, these infections are not transmitted through casual contact and are not contagious in the same way that infectious diseases are.	130	11.2
*Does my attitude—positive or negative—determine my risk of getting or surviving cancer?*	To date, there is no convincing scientific evidence that links a person’s “attitude” to their risk of developing or dying from cancer. If you have cancer, it’s normal to feel sad, angry, or discouraged sometimes and positive or upbeat at other times. People with a positive attitude may be more likely to maintain social connections and stay active, and physical activity and emotional support may help you cope with your cancer. For more information, see the NCI fact sheet on Stress and Cancer.	83	11.2	It is not clear whether having a positive or negative attitude directly affects the risk of developing cancer or the likelihood of surviving cancer. While it is important to maintain a positive outlook and to have a support system during cancer treatment, there is no scientific evidence to suggest that a positive attitude can prevent cancer or improve survival. Cancer is a complex disease that is influenced by a variety of factors, including genetics, environmental exposures, and lifestyle habits. While it is important to take care of your overall health and to adopt healthy habits that may reduce your risk of cancer, it is not possible to control all of the factors that may influence your risk of developing cancer. It is also important to recognize that cancer and its treatment can be physically and emotionally challenging. It is normal to experience a range of emotions during this time, and it is important to take care of your mental health and seek support if you need it.	167	20.2
*Can cancer surgery or a tumor biopsy cause cancer to spread in the body?*	The chance that surgery will cause cancer to spread to other parts of the body is extremely low. Following standard procedures, surgeons use special methods and take many steps to prevent cancer cells from spreading during biopsies or surgery to remove tumors. For example, if they must remove tissue from more than one area of the body, they use different surgical tools for each area. For information about how cancer spreads in the body, see our page on Metastatic Cancer.	80	11.0	In most cases, cancer surgery or a tumor biopsy will not cause cancer to spread in the body. In fact, these procedures are typically done to remove or diagnose cancer that is already present in the body. During surgery, the goal is to remove as much of the cancer as possible, including any visible tumors and any cancer cells that may have spread to nearby tissues. A biopsy is a procedure in which a small sample of tissue is removed and examined under a microscope to determine if cancer is present. In some cases, it is possible that cancer cells may be released into the body during surgery or a biopsy. This can occur when cancer cells are present in the fluids or tissues surrounding the tumor. However, it is important to note that these cells are usually present in very low numbers and are not likely to cause the cancer to spread. It is important to work with a healthcare team to determine the best treatment approach for your situation. This may include surgery, chemotherapy, radiation, or other treatments, depending on the type and stage of your cancer. Your healthcare team can provide more information about the potential risks and benefits of these treatments and help you make the best decision for your situation.	215	13.4
*Will cancer get worse if exposed to air?*	No. Exposure to air will not make tumors grow faster or cause cancer to spread to other parts of the body. For information about how cancer spreads in the body, see our page on Metastatic Cancer.	36	8.8	Exposing cancer to air will not cause it to get worse. Cancer is a disease in which abnormal cells grow and divide uncontrollably, forming tumors. These abnormal cells are present in the body, and they do not require air to grow or spread. It is important to note that cancer surgery may involve exposing cancerous tumors to air. During surgery, the goal is to remove as much of the cancer as possible, including any visible tumors and any cancer cells that may have spread to nearby tissues. In some cases, the cancer may be removed by cutting into the skin, which can expose the cancerous tissue to air. In other cases, the cancer may be removed through laparoscopic surgery, which involves making small incisions in the skin and inserting instruments and a camera through the incisions. It is important to work with a healthcare team to determine the best treatment approach for your situation. This may include surgery, chemotherapy, radiation, or other treatments, depending on the type and stage of your cancer. Your healthcare team can provide more information about the potential risks and benefits of these treatments and help you make the best decision for your situation.	198	12.8
*Do cell phones cause cancer?*	No, not according to the best studies completed so far. Cancer is caused by genetic mutations, and cell phones emit a type of low-frequency energy that does not damage genes. For more information, see the NCI fact sheet on Cell Phones and Cancer Risk.	44	7.5	There is no clear evidence that cell phones or other wireless devices cause cancer. Some studies have suggested a possible link between the use of cell phones and an increased risk of certain types of cancer, such as brain cancer. However, other studies have not found such a link. The World Health Organization (WHO) has classified radiofrequency (RF) fields, which are emitted by cell phones and other wireless devices, as possibly carcinogenic to humans. However, this classification is based on limited evidence from studies of high RF field exposure in animals, and it does not mean that these fields definitely cause cancer. The WHO has also noted that the levels of RF fields emitted by cell phones are typically much lower than those that have been associated with increased cancer risk in animal studies. More research is needed to understand the potential health effects of RF fields and the use of cell phones. In the meantime, it is generally recommended to take steps to reduce your RF field exposure, such as using hands-free devices or texting instead of talking on the phone, and keeping your phone away from your body when it is not in use.	196	15.3
*Do power lines cause cancer?*	No, not according to the best studies completed so far. Power lines emit both electric and magnetic energy. The electric energy emitted by power lines is easily shielded or weakened by walls and other objects. The magnetic energy emitted by power lines is a low-frequency form of radiation that does not damage genes. For more information, see the NCI fact sheet on Electromagnetic Fields and Cancer.	66	13.2	There is no clear evidence that living near power lines causes cancer. Some studies have suggested a possible link between living near power lines and an increased risk of certain types of cancer, such as leukemia and brain cancer. However, other studies have not found such a link. Power lines emit extremely low frequency (ELF) electromagnetic fields (EMFs), which are a type of non-ionizing radiation. Non-ionizing radiation is generally considered to be less harmful than ionizing radiation, which is known to cause DNA damage and increase the risk of cancer. The levels of ELF EMFs emitted by power lines are typically much lower than the levels that have been associated with increased cancer risk in animal studies. More research is needed to understand the potential health effects of ELF EMFs and the risk of cancer. In the meantime, it is generally recommended to take steps to reduce your exposure to all types of EMFs, including those emitted by power lines. This may include using shielding materials to block EMFs, and keeping a safe distance from sources of EMFs when possible.	180	13.7
*Are there herbal products that can cure cancer?*	No. Although some studies suggest that alternative or complementary therapies, including some herbs, may help patients cope with the side effects of cancer treatment, no herbal products have been shown to be effective for treating cancer. In fact, some herbal products may be harmful when taken during chemotherapy or radiation therapy because they may interfere with how these treatments work. Cancer patients should talk with their doctor about any complementary and alternative medicine products—including vitamins and herbal supplements—they may be using. For more information, see Complementary and Alternative Medicine.	91	15.0	There are no herbal products that have been proven to cure cancer. While some herbs and dietary supplements may have potential health benefits, there is limited scientific evidence to support the use of these products as a treatment for cancer. It is important to note that the use of unproven or untested treatments, including herbal products, can be dangerous and may cause serious harm. Some unproven treatments may contain harmful ingredients, may interfere with proven cancer treatments, or may delay the use of effective treatments. If you are considering using an herbal product or other alternative treatment for cancer, it is important to speak with your healthcare provider first. Your healthcare provider can help you understand the potential risks and benefits of these treatments and can help you make an informed decision about your care. It is always a good idea to seek guidance from a qualified healthcare provider before starting any new treatment.	154	15.5
*If someone in my family has cancer, am I likely to get cancer, too?*	Not necessarily. Cancer is caused by harmful changes (mutations) in genes. Only about 5 to 10% of cancers are caused by harmful mutations that are inherited from a person’s parents. In families with an inherited cancer-causing mutation, multiple family members will often develop the same type of cancer. These cancers are called “familial” or “hereditary” cancers. The remaining 90 to 95% of cancers are caused by mutations that happen during a person’s lifetime as a natural result of aging and exposure to environmental factors, such as tobacco smoke and radiation. These cancers are called “non-hereditary” or “spontaneous” cancers. For more information about the risk of getting cancer, see the NCI fact sheet on Genetic Testing for Inherited Cancer Susceptibility Syndromes and Cancer Causes and Risk Factors.	128	13.5	Having a family member with cancer may increase your risk of developing cancer, but it does not guarantee that you will develop the disease. The risk of developing cancer can be influenced by a variety of factors, including genetics, environmental exposures, and lifestyle habits. If you have a family member with cancer, it is important to be aware of your personal risk factors and to take steps to reduce your risk of cancer. This may include adopting healthy habits such as not smoking, maintaining a healthy weight, and exercising regularly. It may also be helpful to speak with your healthcare provider about your family medical history and any recommended screening tests or preventive measures. It is also important to remember that cancer is a complex disease, and not all cases are caused by genetics. Many people who develop cancer do not have a family history of the disease, and conversely, some people who have a family history of cancer may never develop the disease.	164	17.4
*If no one in my family has had cancer, does that mean I’m risk-free?*	No. Based on the most recent data, about 38% of men and women will be diagnosed with cancer at some point during their lives. Most cancers are caused by genetic changes that occur throughout a person’s lifetime as a natural result of aging and exposure to environmental factors, such as tobacco smoke and radiation. Other factors, such as what kind of food you eat, how much you eat, and whether you exercise, may also influence your risk of developing cancer. For more information, see Cancer Causes and Risk Factors.	90	11.8	Having a family history of cancer does not guarantee that you will develop the disease, and not having a family history does not mean that you are risk-free. While genetics can play a role in the development of cancer, there are many other factors that can influence your risk of the disease, including environmental exposures, lifestyle habits, and other medical conditions. It is important to be aware of your personal risk factors for cancer and to take steps to reduce your risk. This may include adopting healthy habits such as not smoking, maintaining a healthy weight, and exercising regularly. It is also important to follow recommended screening guidelines for cancer, which can help detect the disease at an early stage when it is most treatable. If you have concerns about your risk of cancer or have any symptoms that you are concerned about, it is always a good idea to speak with your healthcare provider for guidance.	157	18.7
*Do antiperspirants or deodorants cause breast cancer?*	No. The best studies so far have found no evidence linking the chemicals typically found in antiperspirants and deodorants with changes in breast tissue. For more information, see the NCI fact sheet on Antiperspirants/Deodorants and Breast Cancer.	38	12.6	There is no clear evidence that the use of antiperspirants or deodorants causes breast cancer. Some studies have suggested a possible link between the use of these products and an increased risk of breast cancer, but the results of these studies have been mixed and more research is needed to understand the relationship between antiperspirants and deodorants and breast cancer. The National Cancer Institute (NCI) states that there is currently no strong evidence to support a link between the use of antiperspirants or deodorants and breast cancer. The NCI notes that while some studies have suggested a possible link between the use of these products and an increased risk of breast cancer, the results of these studies have been inconclusive and more research is needed to understand the relationship between these products and breast cancer. If you are concerned about the safety of antiperspirants or deodorants, it is always a good idea to speak with your healthcare provider for guidance. Your healthcare provider can provide more information about the potential risks and benefits of these products and can help you make an informed decision about your personal care.	188	21.8

a FKG = Flesch-Kincaid grade; NCI = National Cancer Institute; WC = word count.

This brief report offers important insights into the potentially positive capabilities of ChatGPT and other AI systems in the context of cancer-related (mis)information. As a team, we were initially uncertain about the utility of ChatGPT for health information and thought that the AI system could unintentionally convey misinformation or harmful information to users. This possibility is still important to consider for any AI chat system, given that users can problematically shape chatbot responses in a short period (6); researchers have found that AI systems can “enact malignant stereotypes” (7); and, specific to ChatGPT, there is reasonable skepticism about the tool’s utility (8) when considering a breadth of topics beyond the basic cancer information we asked about in our study. ChatGPT could be the future of AI chat technology (or perhaps the tool is just a popular example of the state of the technology’s capabilities); regardless of this particular tool’s features, however, we must consider how best to monitor and evaluate the use of these tools in the online communication environment. Future systematic work to evaluate use and outputs of such platforms requires an infrastructure to monitor these sites, their algorithms and information sources, and potential bias in providing information equitably to diverse populations. There is concern that algorithms reinforce current health disparities and inequities (9), although the extent to which this is true about health information specifically is currently unknown. Other practical considerations about how people will interpret AI-generated chat responses in terms of trust and credibility (10) exist, particularly as they relate to medical and health information, behaviors, and services compared with consumer products, behaviors, and services.

The limitations of this study are that we evaluated only common cancer misinformation in the English language. More obscure cancer myths may have incomplete or English-only information, resulting in the model’s inability to be trained on a sufficiently large and diverse data set to answer fewer common questions or non–English-language questions accurately. Finally, although this study obtained contemporary ChatGPT outputs, there is a possibility that these outputs were trained on models that could be scientifically outdated. Currently, ChatGPT is limited to data collected before 2021, so it is possible that as new scientific information emerges, ChatGPT may not be an accurate source of information or, at the least, its accuracy will be delayed.

Collectively, we found ChatGPT outputs in response to common cancer misinformation to be accurate and similar to answers that the NCI provided. In the context of these questions, there does not appear to be a clear area where this system may be susceptible to misinformation. Future research is needed to determine whether other chatbots and AI-driven systems provide accurate cancer information consistently, whether these findings apply to more diverse claims about cancer across the continuum, and what the ideal infrastructure might be for future monitoring to ensure accuracy of cancer information within the online information ecosystem.

## Supplementary Material

pkad015_Supplementary_Data

## Data Availability

The data underlying this article are available online, and the data sets were derived from sources in the public domain: https://chat.openai.com and https://www.cancer.gov/about-cancer/causes-prevention/risk/myths.
