# Supplementary material for: Using ChatGPT to evaluate cancer myths and misconceptions: artificial intelligence and cancer information
Source: JNCI Cancer Spectr. 2023 Mar 17;7(2):pkad015. doi: 10.1093/jncics/pkad015 (PMC10020140; doi:10.1093/jncics/pkad015)
Supplement: pkad015_Supplementary_Data [file pkad015_supplementary_data.docx]

| Supplementary Table 1. Repeated cancer myth/misconception input with subsequent outputs | | | | | |
| --- | --- | --- | --- | --- | --- |
| Input | Output 1 | Output 2 | Output 3 | Output 4 | Output 5 |
| Is cancer a death sentence? | No, cancer is not always a death sentence. While it can be a serious and life-threatening condition, many people with cancer are able to successfully manage their illness and live full and productive lives with appropriate treatment and care. Advances in medical technology and cancer treatments have led to improved survival rates and a growing number of cancer survivors. It's important to remember that each person's experience with cancer is unique and depends on many factors, including the type and stage of the cancer, overall health, and access to quality medical care. | No, cancer is not always a death sentence. While it can be a serious and life-threatening condition, many people with cancer are able to successfully manage their illness and live full and productive lives with appropriate treatment and care. Advances in medical technology and cancer treatments have led to improved survival rates and a growing number of cancer survivors. It's important to remember that each person's experience with cancer is unique and depends on many factors, including the type and stage of the cancer, overall health, and access to quality medical care. | No, cancer is not always a death sentence. Many people with cancer are able to successfully manage their illness and live full and productive lives with appropriate treatment and care. Advances in medical technology and cancer treatments have led to improved survival rates and a growing number of cancer survivors. However, the outcome for each person with cancer will depend on several factors, including the type and stage of the cancer, overall health, and access to quality medical care. | No, cancer is not always a death sentence. While it can be a serious and life-threatening condition, many people with cancer are able to successfully manage their illness and live full and productive lives with appropriate treatment and care. Advances in medical technology and cancer treatments have led to improved survival rates and a growing number of cancer survivors. The outcome for each person with cancer will depend on several factors, including the type and stage of the cancer, overall health, and access to quality medical care. | No, cancer is not a death sentence for everyone. With early detection and appropriate treatment, many people with cancer are able to successfully manage their illness and live full and productive lives. The outcome for each person with cancer will depend on several factors, including the type and stage of the cancer, overall health, and access to quality medical care. While cancer can be a serious and life-threatening condition, advances in medical technology and cancer treatments have led to improved survival rates and a growing number of cancer survivors. |
| Will eating sugar make my cancer worse? | There is no scientific evidence to support the claim that eating sugar directly causes cancer to worsen. However, a diet high in sugar and processed foods can lead to weight gain and obesity, which are associated with an increased risk of certain types of cancer. It is important to maintain a balanced and healthy diet as part of a comprehensive approach to cancer management and treatment. | There is no scientific evidence to support the idea that sugar consumption directly causes cancer to worsen. However, consuming a diet high in sugar and processed foods can contribute to weight gain and obesity, which are associated with an increased risk of certain types of cancer. It is important to maintain a balanced and healthy diet as part of a comprehensive approach to cancer management and treatment. | There is no direct link between sugar consumption and worsening of cancer. However, a diet high in sugar and processed foods can lead to weight gain and obesity, which have been associated with an increased risk of certain types of cancer. To support overall health and well-being, it is recommended to maintain a balanced and nutritious diet, in consultation with a healthcare provider. | No, there is no scientific evidence to suggest that eating sugar directly causes cancer to worsen. However, a diet high in sugar and processed foods can contribute to weight gain and obesity, which are associated with an increased risk of certain types of cancer. To support your overall health and well-being, it's important to maintain a balanced and nutritious diet, in consultation with a healthcare provider. | No, consuming sugar does not directly make cancer worse. However, a diet high in sugar and processed foods can lead to weight gain and obesity, which have been associated with an increased risk of certain types of cancer. It's important to maintain a balanced and nutritious diet to support overall health and well-being, in consultation with a healthcare provider. |
| Do artificial sweeteners cause cancer? | There is currently no strong scientific evidence to support the claim that artificial sweeteners cause cancer. The artificial sweeteners approved for use by regulatory agencies such as the US Food and Drug Administration (FDA) and the European Food Safety Authority (EFSA) are considered safe for consumption in moderate amounts. However, some studies have suggested a potential link between artificial sweetener consumption and an increased risk of certain cancers, but more research is needed to confirm these findings. It's always best to consult a healthcare professional for personalized advice on dietary choices. | There is no definitive evidence that artificial sweeteners cause cancer. The available scientific evidence suggests that artificial sweeteners are safe to consume in moderate amounts. However, some studies have suggested a potential link between the consumption of artificial sweeteners and an increased risk of certain cancers, but more research is needed to confirm these findings. As with all things related to health, it's important to speak with a healthcare professional for personalized advice. | Artificial sweeteners have been widely studied and the current scientific consensus is that they do not cause cancer. Many regulatory agencies, including the US Food and Drug Administration (FDA) and the European Food Safety Authority (EFSA), consider artificial sweeteners to be safe for consumption in moderate amounts. However, some studies have suggested a potential link between artificial sweetener consumption and cancer, but more research is needed to establish a definitive connection. It's always best to consult with a healthcare professional for personalized advice on dietary choices. | There is no conclusive evidence that artificial sweeteners cause cancer. While some studies have suggested a potential link between artificial sweetener consumption and an increased risk of cancer, the weight of scientific evidence does not support this claim. The US Food and Drug Administration (FDA) and other regulatory agencies have deemed artificial sweeteners to be safe for consumption in moderate amounts. It's important to note that more research is needed to establish a definitive connection between artificial sweeteners and cancer. As with all dietary decisions, it's best to consult with a healthcare professional for personalized advice. | There is no clear evidence that artificial sweeteners cause cancer. Most regulatory agencies, such as the US Food and Drug Administration (FDA) and the European Food Safety Authority (EFSA), consider artificial sweeteners to be safe for consumption in moderate amounts. However, some studies have suggested a potential link between artificial sweetener consumption and an increased risk of certain cancers, but more research is needed to establish a definitive connection. It's always best to consult with a healthcare professional for personalized advice on dietary choices. |
| Is cancer contagious? | No, cancer is not contagious. It is a disease caused by the abnormal growth of cells in the body, which can be triggered by genetic mutations, environmental factors, lifestyle choices, or a combination of these factors. It cannot be spread from person to person like a contagious disease. | No, cancer is not contagious. It is caused by abnormal growth of cells in the body, which can be triggered by genetic mutations, environmental factors, lifestyle choices, or a combination of these factors. It cannot be spread from one person to another like a contagious disease. | No, cancer is not contagious. It is a disease caused by abnormal growth of cells in the body, which can be the result of various factors such as genetic mutations, environmental exposures, lifestyle choices, or a combination of these. Cancer cannot be spread from person to person like a contagious disease. | No, cancer is not contagious and cannot be spread from one person to another like a contagious disease. Cancer is caused by abnormal growth of cells in the body, which can be due to various factors such as genetic mutations, environmental exposures, lifestyle choices, or a combination of these. | No, cancer is not contagious. Cancer is a disease that occurs when there is an uncontrolled growth of abnormal cells in the body. This growth can be caused by various factors, including genetic mutations, environmental exposures, lifestyle choices, or a combination of these. Cancer cannot be spread from person to person like a contagious disease. |
| Does my attitude—positive or negative—determine my risk of getting or surviving cancer? | No, your attitude does not determine your risk of getting or surviving cancer. Risk factors for cancer include age, genetics, lifestyle choices (such as tobacco use, alcohol consumption, and diet), and exposure to certain chemicals and substances. While a positive attitude can be helpful for coping with a cancer diagnosis and treatment, it does not directly impact the development or progression of the disease. It is important to understand that cancer is a complex and multifactorial disease, and there is no single factor that determines one's risk or outcome. | No, your attitude does not determine your risk of getting or surviving cancer. Cancer is a complex disease influenced by a combination of factors such as genetics, lifestyle, environment, and medical history. A positive attitude can have psychological and emotional benefits for a person dealing with cancer, but it does not directly impact the risk of getting or surviving the disease. It is important to focus on preventative measures, early detection, and following a recommended treatment plan under the guidance of a healthcare provider to improve outcomes for those living with cancer. | development and progression of cancer are influenced by various factors including genetics, lifestyle, environment, and medical history. While a positive attitude can improve a person's mental and emotional well-being and help with coping during cancer treatment, it does not directly affect the risk of getting or surviving cancer. It is important to focus on preventative measures, early detection, and following a recommended treatment plan under the guidance of a healthcare provider for the best outcome for those with cancer. | "No, your attitude does not determine your risk of getting or surviving cancer. Cancer is a complex disease influenced by a range of factors such as genetics, lifestyle, environment, and medical history. While a positive attitude can have psychological and emotional benefits for a person dealing with cancer, it does not directly impact the risk of getting or surviving the disease. It is important to focus on preventative measures, early detection, and following a recommended treatment plan under the guidance of a healthcare provider to improve outcomes for those living with cancer. | No, your attitude does not determine your risk of getting or surviving cancer. Cancer is a complex disease influenced by a variety of factors such as genetics, lifestyle, environment, and medical history. While a positive attitude can be helpful for coping with a cancer diagnosis and treatment, it does not directly impact the development or progression of the disease. It is important to focus on preventative measures, early detection, and following a recommended treatment plan under the guidance of a healthcare provider for the best outcome for those with cancer. |
| Can cancer surgery or a tumor biopsy cause cancer to spread in the body? | In some cases, cancer surgery or a tumor biopsy can cause the spread of cancer within the body, but this is relatively rare. During surgery, it is possible for cancer cells to be dislodged and spread to other parts of the body through the bloodstream or lymphatic system. Similarly, a biopsy can cause cancer cells to be dispersed into surrounding tissue. However, the benefits of these procedures usually outweigh the risks, and the likelihood of cancer spread can be minimized through proper surgical techniques. Your doctor will be able to assess the risks and benefits of these procedures and determine if they are appropriate for you. | Surgery and biopsy can, in rare cases, cause the spread of cancer cells in the body. This is because the process of removing or biopsying a tumor can sometimes cause the dislodgement and dissemination of cancer cells. However, the benefits of these procedures, such as obtaining a diagnosis, staging the cancer, and potentially removing all or part of a cancerous growth, often outweigh the risk of cancer spread. Your doctor will be able to advise you on the risks and benefits of these procedures in your specific case. | Cancer surgery or a biopsy can, in rare cases, lead to the spread of cancer cells within the body. During surgery, it is possible for cancer cells to be dislodged and spread to other parts of the body through the bloodstream or lymphatic system. Similarly, a biopsy can cause cancer cells to be dispersed into surrounding tissue. However, the benefits of these procedures, such as obtaining a diagnosis and staging the cancer, typically outweigh the risks, and the likelihood of cancer spread can be minimized through proper surgical techniques. Your doctor will be able to advise you on the risks and benefits of these procedures in your specific case. | Yes, cancer surgery or a tumor biopsy can, in rare cases, cause the spread of cancer within the body. During surgery, it is possible for cancer cells to be dislodged and spread to other parts of the body through the bloodstream or lymphatic system. Similarly, a biopsy can cause cancer cells to be dispersed into surrounding tissue. However, the benefits of these procedures, such as obtaining a diagnosis and staging the cancer, typically outweigh the risks, and the likelihood of cancer spread can be minimized through proper surgical techniques. Your doctor will be able to advise you on the risks and benefits of these procedures in your specific case. | It is possible for cancer surgery or a tumor biopsy to cause the spread of cancer cells within the body, although it is a relatively rare occurrence. The risk of cancer spread during surgery can be minimized through proper surgical techniques, and the benefits of these procedures, such as obtaining a diagnosis and potentially removing all or part of a cancerous growth, typically outweigh the risk. A biopsy can also cause cancer cells to spread, but it is often necessary for obtaining a diagnosis and determining the best course of treatment. Your doctor will be able to advise you on the risks and benefits of these procedures in your specific case. |
| Will cancer get worse if exposed to air? | No, exposure to air does not directly cause cancer to worsen. Cancer is a disease caused by the uncontrolled growth of abnormal cells in the body. Exposure to air will not directly affect the progression of cancer. However, some cancer treatments, such as surgery, can cause a cancerous lesion to be exposed to air. In these cases, proper wound care and protection are important to prevent infections and promote healing. | No, exposure to air does not directly cause cancer to worsen. Cancer is a disease that occurs when cells in the body grow and divide abnormally, forming a mass called a tumor. Exposure to air does not directly affect the growth or spread of cancer. However, in some cases, such as after surgical removal of a tumor, the area around the removed tissue may be exposed to air and be at risk for infection. It is important to take proper wound care measures to prevent infections and promote healing in these cases. | No, exposure to air does not make cancer worse. Cancer is a disease that occurs when cells in the body grow and divide abnormally, forming a mass called a tumor. The progression of cancer is not influenced by exposure to air. However, in some cases, such as after surgical removal of a tumor, the area around the removed tissue may be exposed to air and be at risk for infection. Proper wound care and protection is important in these cases to prevent infections and promote healing. | No, exposure to air does not cause cancer to worsen. Cancer is a complex disease that occurs when cells in the body grow and divide abnormally, forming a mass called a tumor. The progression of cancer is not directly influenced by exposure to air. However, if a cancerous tissue is surgically removed and the wound is exposed to air, there may be a risk of infection. Proper wound care and protection are important in these cases to prevent infections and promote healing. | No, exposure to air does not make cancer worse. Cancer is a disease that occurs when cells in the body grow and divide abnormally, forming a mass called a tumor. The progression of cancer is not influenced by exposure to air. However, in some cases, such as after surgical removal of a tumor, the area around the removed tissue may be exposed to air and be at risk for infection. It is important to take proper wound care measures to prevent infections and promote healing in these cases. |
| Do cell phones cause cancer? | There is currently no scientific evidence that definitively proves that cell phone usage causes cancer. However, some studies have suggested a possible link between long-term cell phone usage and certain types of cancer, such as brain cancer. More research is needed to determine the potential health effects of cell phone use. In the meantime, it is recommended to take precautions, such as using hands-free devices or texting instead of making calls, to minimize exposure to radiofrequency energy from cell phones. | There is no conclusive evidence that cell phones cause cancer. Some studies have suggested a possible link between long-term cell phone use and certain types of cancer, such as brain cancer, but more research is needed to determine the potential health effects of cell phone use. It is important to note that the level of radiofrequency energy emitted by cell phones is generally low and considered safe by international health agencies. Nevertheless, it is recommended to take precautions, such as using hands-free devices or texting instead of making calls, to minimize exposure to radiofrequency energy from cell phones. | The current scientific evidence does not definitively prove that cell phones cause cancer. While some studies have suggested a potential link between long-term cell phone use and certain types of cancer, such as brain cancer, more research is needed to determine the potential health effects of cell phone use. The levels of radiofrequency energy emitted by cell phones are generally low and considered safe by international health agencies. However, it is recommended to take precautions, such as using hands-free devices or texting instead of making calls, to minimize exposure to radiofrequency energy from cell phones. | At this time, there is no strong scientific evidence to support a direct causal link between cell phone use and cancer. Some studies have suggested a potential association, particularly between long-term cell phone use and certain types of brain cancer, but more research is needed to establish a definitive connection. It's also worth noting that the levels of radiofrequency energy emitted by cell phones are generally low and considered safe by international health agencies. Despite this, it's still recommended to take precautions to minimize exposure, such as using hands-free devices or texting instead of making calls. | The relationship between cell phone use and cancer is still being studied and is not yet fully understood. While some studies have suggested a possible link between long-term cell phone use and certain types of cancer, such as brain cancer, more research is needed to establish a definite connection. Currently, the levels of radiofrequency energy emitted by cell phones are considered safe by international health agencies. However, as a precaution, it is recommended to minimize exposure by using hands-free devices or texting instead of making calls. |
| Do power lines cause cancer? | There is no clear evidence that exposure to the electromagnetic fields (EMF) generated by power lines causes cancer. Some studies have suggested a possible link between exposure to high levels of EMF and an increased risk of certain types of cancer, such as leukemia, but the evidence is not consistent and more research is needed to establish a definitive connection. The World Health Organization and other health agencies have classified EMF as a "possible carcinogen," but it is important to note that this classification is based on limited evidence and more research is needed to fully understand the potential health effects of EMF exposure. | There is limited evidence to suggest that exposure to the electromagnetic fields (EMF) generated by power lines may increase the risk of certain types of cancer, such as leukemia. However, the evidence is not consistent and more research is needed to establish a definitive connection. The World Health Organization classifies EMF as a "possible carcinogen," but this classification is based on limited evidence. It is important to note that the levels of EMF exposure from power lines are generally low and do not pose a significant health risk to the general population. | There is ongoing scientific debate about the potential health effects of exposure to the electromagnetic fields (EMF) generated by power lines. While some studies have suggested a link between exposure to high levels of EMF and an increased risk of certain types of cancer, such as leukemia, the evidence is not consistent and more research is needed to establish a definitive connection. The World Health Organization classifies EMF as a "possible carcinogen," based on limited evidence, but other health agencies have not found a clear link between EMF exposure and cancer. It is important to note that the levels of EMF exposure from power lines are generally low and do not pose a significant health risk to the general population. | The relationship between power lines and cancer is still a subject of scientific debate and research. Some studies have suggested a link between exposure to high levels of electromagnetic fields (EMF) generated by power lines and an increased risk of certain types of cancer, such as leukemia. However, the evidence is not consistent and more research is needed to establish a clear and definitive connection. The World Health Organization classifies EMF as a "possible carcinogen," based on limited evidence, but it is important to note that the levels of EMF exposure from power lines are generally low and do not pose a significant health risk to the general population. | The scientific evidence for a connection between power lines and cancer is limited and inconclusive. Some studies have suggested a link between exposure to high levels of electromagnetic fields (EMF) generated by power lines and an increased risk of certain types of cancer, such as leukemia. However, the evidence is not consistent and more research is needed to establish a clear and definitive connection. The World Health Organization classifies EMF as a "possible carcinogen," but this classification is based on limited evidence. It is important to note that the levels of EMF exposure from power lines are generally low and do not pose a significant health risk to the general population. |
| Are there herbal products that can cure cancer? | No, there are currently no herbal products that have been scientifically proven to cure cancer. While some herbs and supplements may have potential benefits for managing the symptoms of cancer or side effects of cancer treatments, they should not be relied upon as a substitute for conventional medical treatment such as surgery, chemotherapy, and radiation therapy. It's important to talk to your doctor before taking any new supplements or making significant changes to your diet, as some herbs can interact with cancer treatments or have adverse effects. | No, there is no scientific evidence to support the claim that any herbal product can cure cancer. Cancer is a complex disease that requires medical treatment such as surgery, chemotherapy, and radiation therapy. While some herbal products may have potential benefits for managing the symptoms of cancer or side effects of cancer treatments, they should not be used as a substitute for conventional medical treatment. It is important to talk to a doctor before taking any herbal products or making significant changes to your diet, as some herbs can interact with cancer treatments or have adverse effects. | No, there is no herbal product that has been scientifically proven to cure cancer. Cancer is a complex disease that requires medical treatment such as surgery, chemotherapy, and radiation therapy. While some herbal products may have potential benefits for managing the symptoms of cancer or side effects of cancer treatments, they should not be used as a substitute for conventional medical treatment. It's important to consult with a doctor before taking any herbal products or making significant changes to your diet, as some herbs can interact with cancer treatments or have adverse effects. | No, there is no scientific evidence that any herbal product can cure cancer. Cancer is a complex disease that requires medical treatment such as surgery, chemotherapy, and radiation therapy. While some herbal products may have potential benefits for managing the symptoms of cancer or side effects of cancer treatments, they should not be relied upon as a substitute for conventional medical treatment. It's important to talk to your doctor before taking any herbal products or making significant changes to your diet, as some herbs can interact with cancer treatments or have adverse effects. | No, there is no scientific evidence to support the claim that any herbal product can cure cancer. Cancer is a complex disease that requires medical treatment such as surgery, chemotherapy, and radiation therapy. While some herbal products may have potential benefits for managing the symptoms of cancer or side effects of cancer treatments, they should not be used as a substitute for conventional medical treatment. It's important to talk to a doctor before taking any herbal products or making significant changes to your diet, as some herbs can interact with cancer treatments or have adverse effects. |
| If someone in my family has cancer, am I likely to get cancer, too? | Having a family member with cancer can increase your risk of developing cancer, but it does not guarantee it. There are many factors that contribute to an individual's risk of cancer, including genetics, lifestyle, and environmental factors. If you have a family history of cancer, it is recommended to speak with a doctor who can assess your risk and recommend any necessary screenings or lifestyle changes. | Having a family member with cancer can increase your risk of developing cancer, but it does not guarantee it. There are many factors that contribute to an individual's risk of cancer, including genetics, lifestyle, and environmental factors. If you have a family history of cancer, it is recommended to speak with a doctor who can assess your risk and recommend any necessary screenings or lifestyle changes. | Having a family member with cancer can increase your risk of developing the disease, but it does not guarantee it. The likelihood of developing cancer is influenced by a combination of genetic and environmental factors. If you have a family history of cancer, it is important to discuss your risk with a doctor, who can recommend any necessary screenings or lifestyle changes to help reduce your risk. It's also important to maintain a healthy lifestyle, including a balanced diet and regular physical activity, to help reduce your risk of cancer. | The risk of developing cancer can be increased if a family member has been diagnosed with the disease, but it does not necessarily mean that you will also develop cancer. Many factors, including genetics, lifestyle, and environmental exposure, can influence an individual's risk of cancer. If you have a family history of cancer, it is important to talk to your doctor, who can help assess your personal risk and recommend any necessary screenings or preventative measures. However, it's important to remember that having a family history of cancer does not guarantee that you will develop the disease. | Having a family member with cancer can increase your risk of developing the disease, but it does not guarantee it. The likelihood of developing cancer is influenced by a combination of genetic and environmental factors. If you have a family history of cancer, it is important to discuss your risk with a doctor, who can recommend any necessary screenings or lifestyle changes to help reduce your risk. Maintaining a healthy lifestyle, such as eating a balanced diet and getting regular exercise, can also help reduce your risk of cancer. It's important to remember that having a family history of cancer does not mean that you will definitely develop the disease, and many people with a family history of cancer never develop the disease themselves. |
| If no one in my family has had cancer, does that mean I’m risk-free? | No, having no family history of cancer does not guarantee that you are risk-free. Cancer can be caused by a combination of genetic and environmental factors, and just because your family does not have a history of it does not mean you are immune. It is important to maintain a healthy lifestyle and undergo regular check-ups and screenings to catch any potential issues early. | No, having no family history of cancer does not necessarily mean you are risk-free. While family history can be a risk factor for certain cancers, many other factors such as age, lifestyle, and exposure to environmental toxins can also play a role in cancer development. It's important to adopt healthy habits and maintain regular cancer screenings to monitor your health. | No, having no family history of cancer does not guarantee that you are risk-free. While family history can increase the risk for certain types of cancer, other factors such as lifestyle habits, environmental exposure, and chance can also play a role. It's important to maintain a healthy lifestyle, get regular check-ups and screenings, and understand your individual risk factors to help reduce the chance of developing cancer. | No, having no family history of cancer does not mean you are completely risk-free. There are many factors that contribute to the development of cancer, including genetics, lifestyle, environmental exposure, and chance. Even if no one in your family has had cancer, it's still important to maintain a healthy lifestyle, get regular check-ups and screenings, and understand your individual risk factors to help reduce your chance of developing cancer. | No, having no family history of cancer does not guarantee that you are risk-free. There are many factors that can increase the risk of developing cancer, including genetics, lifestyle, environmental exposures, and chance. While having a family history of cancer can increase your risk, it is just one of many factors that should be considered when evaluating your overall cancer risk. It's important to maintain a healthy lifestyle, undergo regular cancer screenings, and understand your individual risk factors to help reduce the chance of developing cancer. |
